# Integrated transcriptomic and proteomic profiling suggests PDK4 and cell cycle-related responses to electroacupuncture in a rat model of chronic atrophic gastritis

**DOI:** 10.3389/fgstr.2026.1894243

**Published:** 2026-10-30

**Authors:** Ling Chen, Ting Qin, Yichen Wang, Ji Wang, Yikun Wu, Xinjing Yang, Yufeng Xie

**Affiliations:** 1 Acupuncture and Moxibustion Department, Shenzhen Hospital (Futian) of Guangzhou University of Chinese Medicine, Shenzhen, China; 2 College of Acupuncture-moxibustion and Tuina, Fujian University of Traditional Chinese Medicine, Fuzhou, China; 3 The Sixth Clinical Medical College, Guangzhou University of Chinese Medicine, Shenzhen, China; 4 School of Medicine, Xiamen University, Xiamen, China

**Keywords:** chronic atrophic gastritis, electroacupuncture, PDK4, proteomics, transcriptomics

## Abstract

**Background:**

Chronic atrophic gastritis (CAG) is a precancerous gastric lesion. Although electroacupuncture (EA) can improve CAG-related symptoms and mucosal injury, its molecular responses remain unclear.

**Methods:**

Male SPF SD rats were assigned to Control, CAG Model, and EA groups. CAG was induced by a multifactorial protocol. EA was applied at Zusanli (ST36) and Zhongwan (CV12) for 20min/day for 2 weeks. Gastric histopathology was evaluated via hematoxylin-eosin staining. To identify candidate targets of EA for CAG, integrated transcriptomics and data-independent acquisition (DIA) proteomics were employed. Subsequent validation was performed by RT-qPCR.

**Results:**

EA significantly attenuated gastric mucosal atrophy and inflammatory cell infiltration, accompanied by reduced CDK4 and CDK6 mRNA levels (*P* < 0.05). Transcriptomics identified 1662 DEGs enriched in estrogen signaling pathway and transcriptional misregulation in cancer signaling pathway. DIA-based proteomic identified 298 DEPs enriched in drug metabolism, amino acid metabolism, muscle contraction, muscle regulation, and mitochondrial metabolism. Integrated analysis identified 24 concordant DEGs/DEPs and RT-qPCR confirmed significant downregulation of PDK4 in the EA group (P<0.01).

**Conclusion:**

EA ameliorated gastric mucosa injury in CAG rats. Downregulation of PDK4 and CDK4/CDK6 mRNA was associated with EA intervention and may contribute to the amelioration of gastric mucosal injury in CAG.

## 1 Introduction

Chronic atrophic gastritis (CAG) is a digestive system disease with a global incidence rate of 10%-30%. As one of the important causes of gastric cancer, CAG is the most common stage in the development of gastric cancer (GC) (1). The progression from normal gastric mucosa→superficial gastritis→CAG→intestinal metaplasia→dysplasia→GC has been widely accepted in the development of GC (2). CAG, representing the early stage of GC development, significantly increases the risk of gastric cancer to 4.12 (3, 4). Therefore, focusing on CAG, as the key initial precursor lesion, is crucial for the primary prevention and early intervention of GC.

In clinical practice, most CAG patients have no obvious symptoms. When symptoms are present, they manifest primarily as dyspepsia, epigastric pain, abdominal distension, and other nonspecific symptoms. ​CAG is caused by various factors, including Helicobacter pylori (H. pylori) infection, bile reflux, advancing age, and unhealthy dietary habits. Furthermore, other contributory mechanisms such as dysbiosis of the gut microbiota (5), genetic variations (6), oxidative stress (7) and immune dysregulation may synergistically promote the disease process.​ The development of CAG can lead to abnormal structure, morphology, and differentiation of gastric mucosal epithelial cells (8). Although some drugs in clinical trials have improved the histopathology and clinical symptoms of CAG patients. Due to the complexity of the pathogenesis of CAG and the difficulty of diagnosing and treating the disease, effective treatment has not yet been developed (9, 10).

Although H. pylori infection is considered the critical trigger of CAG, a recent meta-analysis has shown that patients receiving long-term treatment with the proton pump inhibitor (PPI) omeprazole have a significantly higher incidence of CAG (11), as long-term PPI use may change the colonization mode of H pylori. This could accelerate the process of gland loss and subsequently result in the appearance of atrophic gastritis (12) The relationship between long-term PPI use and the development of CAG is controversial, this has become a dilemma in decision-making concerning the use of PPIs for maintenance treatment, as it has become one of the most commonly used medications worldwide (13) It is important to look for alternative therapies to improve symptoms of CAG patients and improve their quality of life.

Acupuncture has been used to treat CAG in traditional Chinese medicine (TCM) for centuries. A growing number of studies have shown that acupuncture treatment can improve the clinical symptoms of CAG patients with low cost and high safety (14–16). Recent studies have demonstrated unique advantages of acupuncture in ameliorating gastric mucosal damage and regulating immune-metabolic disorders in CAG (17, 18). However, the comprehensive integration of transcriptomic and proteomic approaches in studying acupuncture for CAG remains largely unexplored. To address this gap, our study employed integrated transcriptomic and proteomic analyses to reveal the molecular mechanisms underlying acupuncture’s regulatory effects on CAG.

## 2 Methods

### 2.1 Animals and experimental procedure

Twenty-five specific pathogen-free (SPF) male Sprague-Dawley (SD) rats, aged 6 weeks (250g ± 40g), were housed in standardized laboratory conditions in Xiamen University (Animal Use License No.: SCXK (Hu) 2022-0004). The facility maintained a room temperature of 24-26 °C, relative humidity of 60%-70%, and a natural light-dark cycle. Prior to the experiment, the rats underwent a one-week acclimatization period with free access to food and water. The experimental procedures were performed in accordance with the Guidelines of Care and Use of Laboratory Animals of the Ministry of Science and Technology of the People’s Republic of China, and were approved by the Animal Care and Use Committee of Xiamen University (Ethics Approval No.: XMULAC20230108).

Rats were randomly allocated into three groups: control group (Con, n=7), chronic atrophic gastritis group (Mod, n=9), and electroacupuncture group (EA, n=9) using the random number table method. Except for the Con group, all other two groups underwent the CAG modeling procedure. Following 12 weeks of animal modeling and 2 weeks of EA intervention (from week 10 of modeling for 2 weeks in EA group). Rat body weight was measured weekly. At the end of the 12-week modeling period and after EA intervention, rats underwent a 12-hour fast prior to euthanasia and tissue collection.

A schematic overview of the experimental timeline, including acclimatization, CAG modeling, EA intervention, and tissue collection, is provided in **Supplementary Figure S1**.

### 2.2 CAG modeling

The chronic atrophic gastritis (CAG) model was established by using a multi-factor protocol combining chemical stimulation, physical stress, and irregular feeding over 12 weeks (19). Chemical stimulation involved alternating daily administration of solutions: 0.1% ammonia water replaced normal drinking water for 24 hours on day 1, followed by replacement with 20 mmol/L sodium deoxycholate solution for 24 hours on day 2. The two solutions mentioned above were alternately administered daily for 12 weeks. Concurrently, physical stress was induced daily by applying a large-sized binder clip to the distal one-third of the rat’s tail for 30 minutes per session. Furthermore, an irregular feeding regimen was implemented, consisting of alternating cycles of two days of ad libitum feeding with sufficient standard chow followed by one day of fasting. This modeling process was maintained continuously for 12 weeks.

### 2.3 EA procedure

Zusanli (ST36) and Zhongwan (CV12) were selected for EA intervention, with localization referenced to the Experimental Acupuncture and Moxibustion textbook of the National 13th Five-Year Plan for Higher Education in Traditional Chinese Medicine.

ST 36 is located on the posterolateral aspect of the knee joint, approximately 5mm below the head of the fibula, and CV12 is located approximately 20 mm superior to the umbilicus. Daily EA interventions were administered in the morning. Rats were anesthetized using an inhalation anesthesia machine with 3-5% isoflurane for induction and 1-2% isoflurane for maintenance, then secured in supine position. Disposable acupuncture needles (0.16 mm×13 mm) were inserted into CV12 and ST36 at 3–4 mm depth, followed by additional needle insertions 1 mm lateral to each acupoint. Both needle pairs were connected to an EA apparatus (Hwato SDZ-II, Suzhou Medical Technology Co., Ltd.) with dense-disperse wave at 1/5 Hz and pulse width at 0.2ms ± 20%. Stimulation intensity was set at 1–2 mA, adjusted to elicit mild tremors of the hindlimb and abdominal muscles. EA was applied for 20 minutes daily over two consecutive weeks. Rats in Control group and Model group received equivalent isoflurane anesthesia without EA intervention. Blinding of the investigator administering EA was not feasible because the intervention required knowledge of group allocation.

### 2.4 Sample collection and histopathology

At the end of the 12-week modeling period and EA intervention, rats were fasted for 12 h and then anesthetized with 3-5% isoflurane delivered via an inhalation anesthesia machine. After adequate anesthesia was confirmed, the thoracic and abdominal areas were disinfected with 75% ethanol. Sterilized instruments were used for dissection and sample collection. The abdominal cavity was opened along the midline, and 4–5 mL of blood was collected from the abdominal aorta before tissue harvesting. The stomach was excised and placed in a disposable curved tray. After removing attached soft tissues and adipose, the organ was incised along the greater curvature using surgical scissors. Gastric mucosal surfaces were rinsed with normal saline to remove debris. Five gastric tissue samples were obtained: four were aliquoted into sterile microcentrifuge tubes, flash-frozen in liquid nitrogen, and stored at -80 °C for subsequent analysis; the remaining sample was fixed in 4% paraformaldehyde at room temperature. Upon completion of tissue collection, all animals remained under deep isoflurane anesthesia and were euthanized by exsanguination (via abdominal aorta blood collection). Death was verified by confirmation of cardiac arrest and the loss of pedal withdrawal and corneal reflexes.

Gastric tissues were fixed in 4% paraformaldehyde for 24h, dehydrated through ethanol gradients, cleared in xylene, and paraffin-embedded. Serial 5-μm sections were cut using a rotary microtome, mounted on slides, and dried at 63 °C. Following xylene dewaxing and ethanol rehydration, sections were stained with hematoxylin and eosin (H&E), differentiated, blued, dehydrated through ascending alcohols, cleared in xylene, and coverslipped with neutral resin. Histopathological evaluation of gastric mucosa was performed under light microscopy at 100× magnification.

Three biological replicates per group were used for transcriptomic and proteomic analyses. The mRNA levels of CDK4 and CDK6 were determined with three biological replicates per group, while RT-qPCR validation of PDK4 and SMG1 was conducted using four biological replicates per group. Differences in the number of biological replicates across downstream analyses were attributed to sample availability.

### 2.5 RNA-seq and analysis

#### 2.5.1 RNA extraction and library preparation

Gastric tissues stored at -80 °C were homogenized in TRIzol reagent. Total RNA was extracted through phase separation with chloroform (0.2:1 TRIzol), isopropanol precipitation, and 75% ethanol washes. RNA pellets were air-dried and dissolved in 0.1% DEPC-treated water. Polyadenylated mRNA was enriched by using oligo-dT magnetic beads and fragmented into 300 bp segments. cDNA libraries were constructed via double-stranded cDNA synthesis, end repair, adapter ligation, and PCR amplification using the Illumina NovaSeq Reagent Kit.

#### 2.5.2 Sequencing and bioinformatics

Raw reads were quality-controlled with fastp (https://github.com/OpenGene/fastp) by removing adapters, low-quality bases, and reads with >10% N bases. Clean reads were aligned to the rat reference genome by using HiSAT2, with gene expression quantified as TPM via RSEM. Differential gene expression analysis was performed using DESeq2 with raw count data as input. Differentially expressed genes (DEGs) were identified using P < 0.05 and a fold change (FC) ≥ 1.5 or ≤ 0.67.

#### 2.5.3 Functional and network analysis

DEGs underwent functional enrichment analysis in GO and KEGG databases (p<0.05). Protein-protein interaction (PPI) networks of significant DEGs were constructed using STRING (https://string-db.org/). Weighted network analysis was performed using soft thresholding (pairwise correlation coefficients between genes) with weights ranging from 0.5 to 1. Functional modules with the highest subnetwork scores were selected, and node genes within these modules were scored via the Maximal Clique Centrality (MCC) algorithm plugin implemented in the Cytoscape (v3.10.0). Analyses and figures were conducted on the Majorbio Cloud Platform (https://www.majorbio.com).

### 2.6 Data-independent acquisition (DIA)-based proteomic profiling and analysis

#### 2.6.1 Protein preparation

Total proteins of gastric tissues were extracted from liquid nitrogen-pulverized tissues using RIPA lysis buffer, followed by ultrasonication (4 °C, 30 min) and centrifugation (14,000×g, 15 min, 4 °C). Protein concentration was determined by BCA assay. Aliquots (100 μg) underwent sequential processing: reduction (10 mmol/L TCEP in 10 mmol/L TEAB, 37 °C, 1 h), alkylation (40 mmol/L IAA, RT, 40 min/dark), and acetone precipitation (-20 °C, 4 h). Pellets were digested with trypsin (1:50, 37 °C, 16 h) in 100 mM TEAB. Peptides were desalted via HLB cartridges and quantified by NanoDrop One (Thermo Scientific).

#### 2.6.2 DIA mass spectrometry

The dried peptide sample was redissolved mobile phase A (2%ACN, 0.1% FA)) and B (80%ACN, 0.1% FA) and centrifuged at 20,000 ×g for 8 min. Separation was carried out by a Vanquish Neo liquid chromatograph (Thermo Scientific, USA) with a uPAC High-Throughput column (75μm×5.5cm). The nanoliter liquid phase separation samples were analyzed in DIA mode via an Orbitrap Astral mass spectrometer (Thermo Scientific, USA) operated in positive ionization mode, ion source voltage 1.5 kV, MS scan range 100–1700 m/z. Data acquisition utilized Thermo Xcalibur 4.7.

#### 2.6.3 Data analysis

Raw DIA data were processed using Spectronaut 19 for database searching. Datasets were analyzed on the Majorbio Cloud Platform (https://cloud.majorbio.com). A fold change >1.5 or P value <0.05 (|log_2_FC|≥0.585), and p<0.05 were set as the cutoffs to identify differentially expressed proteins (DEPs). KEGG (Kyoto Encyclopedia of KEGG (Kyoto Encyclopedia of Genes and Genomes) pathway enrichment analysis was performed for DEPs with statistical significance set at p<0.05. Protein-protein interaction (PPI) network analysis was performed using the STRING database (http://string-db.org) and the data were visualized using Cytoscape software (v3.10.0) with the MCODE plugin to identify cluster networks and the cytoHubba plugin to identify hub proteins with the following parameters: degree≥2, remove the single connection node (Haircut), node score threshold = 0.2, k-core = 2 (minimum node degree within subnets), and maximum depth = 100.

### 2.7 Integrated analysis of multi-omics data and validation

Transcriptomic and proteomic profiles were integrated to identify consistently differentially expressed genes/proteins (DEGs/DEPs) based on nine-quadrant classification, and linear regression analysis was performed to assess the relationship between transcript and protein expression levels of the concordant genes/proteins. KEGG pathway enrichment analysis was performed on concordant DEGs and DEPs, comparing enrichment patterns between transcriptomic and proteomic datasets to identify pathways specifically enriched in either omics layer, with focus on CAG-relevant pathways. Key target genes from prioritized signaling pathways were selected based on their association with CAG pathogenesis and validated by RT-qPCR. In addition, CDK4 and CDK6 mRNA levels were also measured by RT-qPCR. CDK4 (Cyclin-dependent kinases 4) and CDK6 (Cyclin-dependent kinases 6) are important regulators of G1/S cell cycle progression, and their transcript levels were measured as cell cycle-related readouts in this study.

### 2.8 Statistical analysis

Data were analyzed by social science statistical software (SPSS) 26 (IBM, Armonk) and GraphPad Prism 5.0 (GraphPad Software Inc.). All the experimental data were expressed as the mean ± standard deviation (SD) unless otherwise indicated. Body weight measurements underwent repeated-measures ANOVA with weeks as within-subject factors and groups as between-subject factors. One-way ANOVA was applied for normally distributed data and non-parametric tests were applied for non-normal distributed data. *Post-hoc* tests (Tukey/Dunnett) were selected based on normality and homogeneity (Levene’s test) results. Significance was considered at *P* < 0.05.

## 3 Results

### 3.1 EA ameliorated gastric lesions and downregulated CDK4 and CDK6 expressions

There was no significant difference in body weights among groups at baseline (*P* > 0.05). After the CAG modeling (week 12), the body weights in the model group and EA group were significantly decreased compared to the control group (*P* < 0.05) as shown in **Figure 1A** and **Supplementary Table 1**.

**Figure 1:**
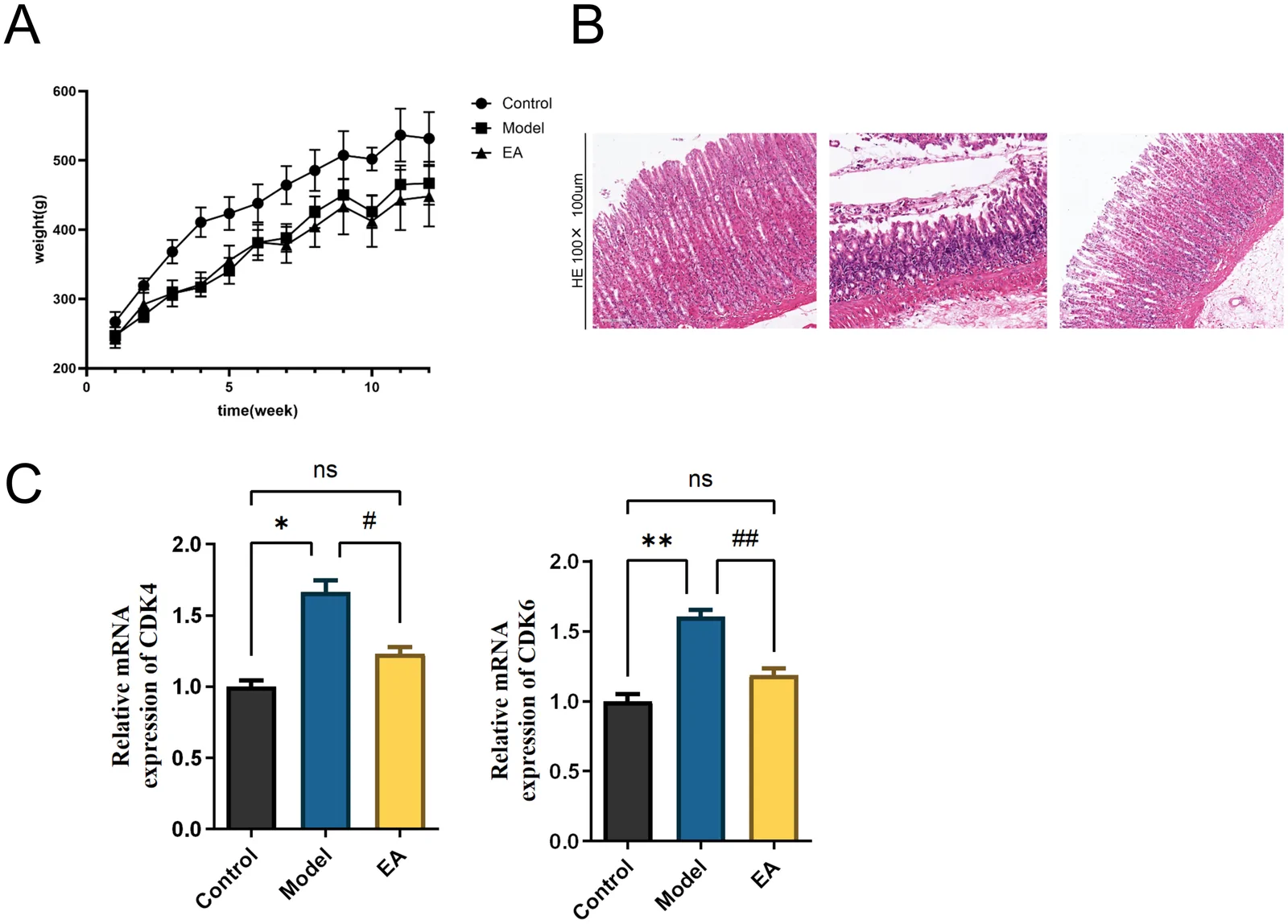
Body weight, histopathology, and expressions of CDK4 and CDK6 in rats. **(A)** Rat body weight was monitored daily (Control, n=7; Model, n=9; EA, n=9). Body weight data were recorded every 7 days for a total of 12 times, and statistical analysis was performed. **(B)** Gastric histopathology of rats was observed by HE staining. **(C)** The mRNA levels of CDK4 and CDK6 in rat gastric tissues were measured by RT-qPCR. ***P<0.05, P<0.01: versus the control group; #P<0.05, ##P<0.01: versus the model group.

As shown in **Figure 1B**, HE staining showed that rats in the control group exhibited intact glandular architecture without inflammation; rats in the model group showed glandular atrophy, disorganization, and dense inflammatory infiltration; and EA-treated rats displayed near-normal mucosal organization with only sparse lymphocytic infiltration.

Compared to the control group, model rats exhibited significantly increased mRNA levels of CDK4 and CDK6. Conversely, EA intervention significantly downregulated CDK4 and CDK6 mRNA levels relative to the model group (*P* < 0.05) (**Appendix Table 1**; **Figure 1C**).

### 3.2 Transcriptomic analysis

#### 3.2.1 Transcriptomic profiling revealed differential gene expression patterns

RNA-seq analysis identified that there were 1665 differentially expressed genes (DEGs) between the model group and the control group (1042 upregulated, 623 downregulated). EA intervention induced 1662 DEGs compared to the model group (809 upregulated, 853 downregulated), and 862 DEGs were identified between EA and control groups (377 upregulated, 485 downregulated). Volcano plots (**Figure 2A**) and hierarchical clustering heatmaps (**Figure 2B**) demonstrated significant differences in transcriptional profiles across groups. The transcriptome sequencing data are shown in the **Supplementary Tables 2–1** and **2–2**.

**Figure 2:**
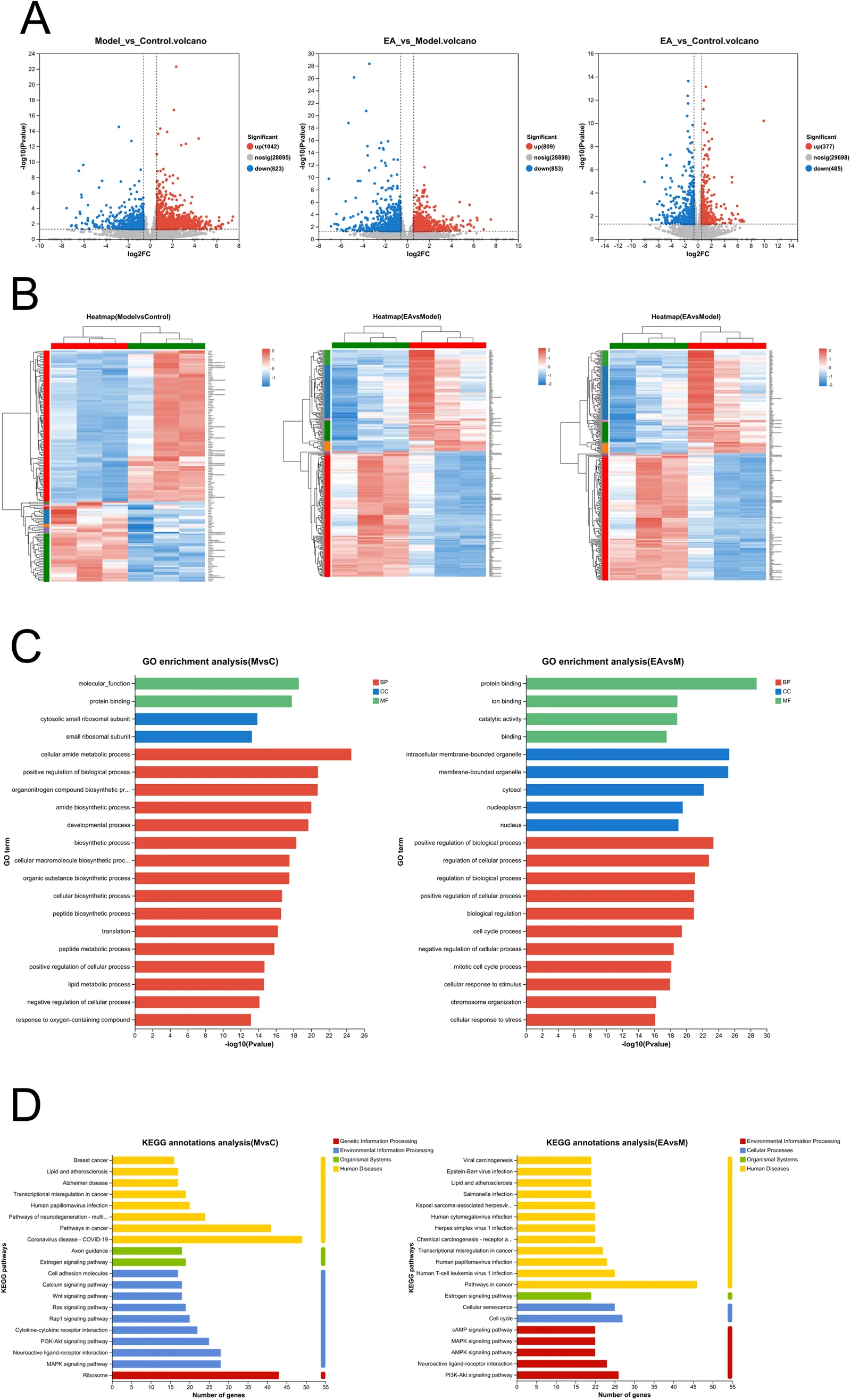
Distribution, clustering, and functional analysis of transcriptomic differentially expressed genes in rat gastric tissues, **(A)** Volcano plots of differentially expressed genes. **(B)** Hierarchical clustering heatmaps of differentially expressed genes. **(C)** Top 20 differentially expressed genes (P < 0.05) visualized by GO enrichment analysis. **(D)** Transcriptomic KEGG annotation analysis between the model and blank control groups, and between the electroacupuncture (EA) and model groups. The results are presented based on the total count of the five selected categories, showing the top 20 items in abundance with P value < 0.05.

#### 3.2.2 GO enrichment and KEGG pathway analysis

GO enrichment and KEGG pathway analysis of DEGs is presented in **Figures 2C, D**, respectively. Bubble plots (**Figure 3A**) display all significantly enriched KEGG pathways (*P* < 0.05). The commonly enriched pathways were identified by taking the intersection of pathway enrichment between the EA vs Model and Model vs Control comparison groups, including: Biosynthesis of unsaturated fatty acids, Breast cancer, Estrogen signaling pathway, Fatty acid elongation, Fluid shear stress and atherosclerosis, Insulin resistance, Transcriptional misregulation in cancer and VEGF signaling pathway.

**Figure 3:**
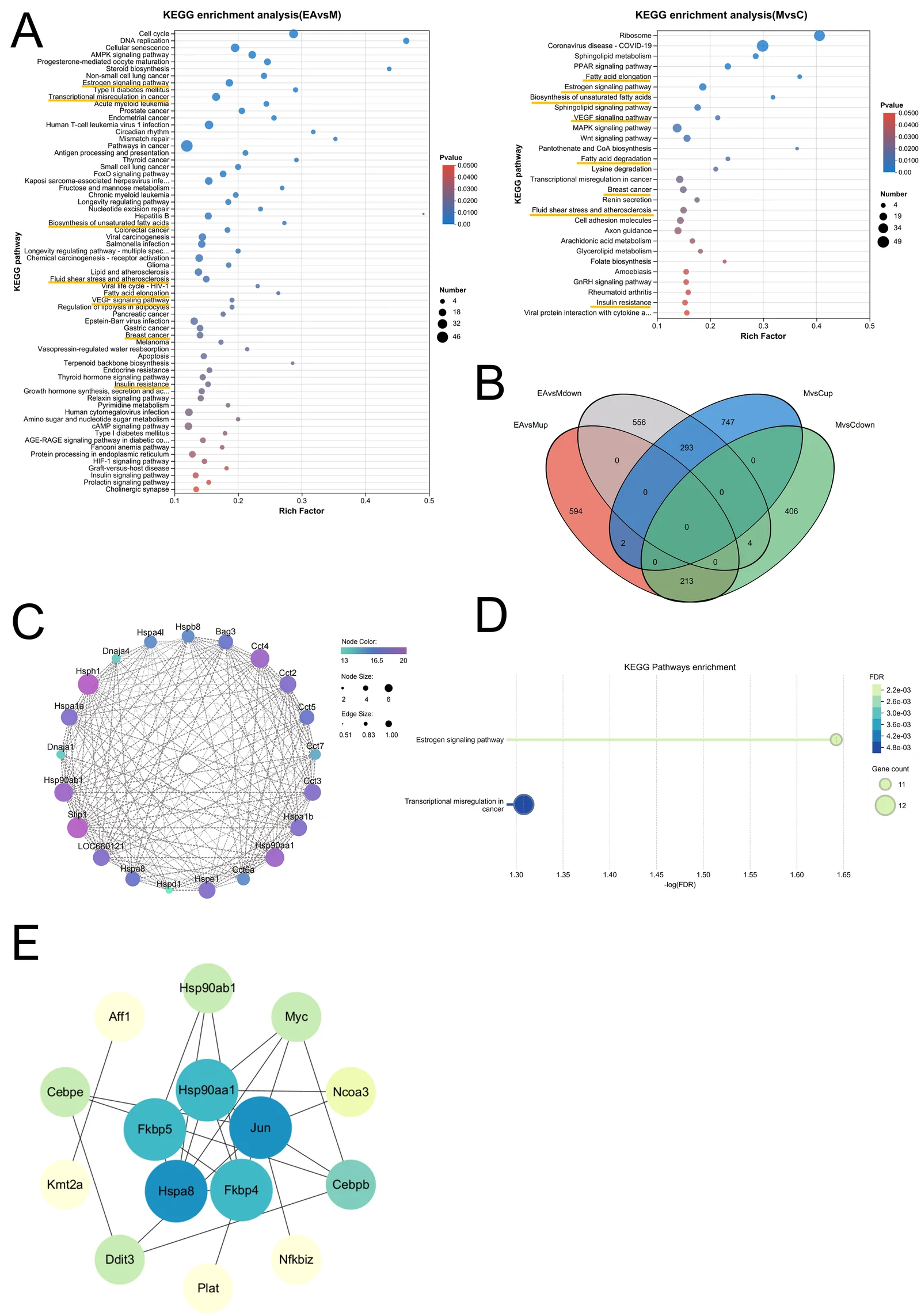
Network profiling of targets for EA-reverted genes. **(A)** KEGG pathway enrichment analysis of differentially expressed genes (DEGs). (P<0.05, underlined pathways are significantly enriched in both EA vs M and M vs C comparisons) **(B)** Venn diagram of differentially expressed genes with reverted expression in EA vs M vs M vs C comparisons **(C)** Enrichment subnetwork: protein folding and chaperone complex **(D)** CytoHubba analysis of reverted genes in EA-regulated core pathways. **(E)** Cytohubba analysis of reversed genes in pathways.

#### 3.2.3 Network profiling of targets for EA-reverted genes

To identify candidate targets of EA, EA-reverted DEGs (compared with the model group) were screened and performed KEGG enrichment and MCODE clustering analysis (**Figure 3B**). STRING analysis generated a protein-protein interaction (PPI) network comprising 448 nodes and 847 edges, with topological metrics: average node degree=3.79, local clustering coefficient=0.392 (PPI enrichment *P* < 1.0×10^-^¹^6^). The highest-scoring cluster (protein folding and chaperone complex) was prioritized for weighted analysis (**Figure 3C**).

KEGG enrichment analysis of reverted DEGs (FDR< 0.05) revealed that core pathways regulated by EA were the estrogen signaling pathway and transcriptional misregulation in cancer signaling pathway (**Figure 3D**). **Table 1** showed DEGs expressions (Model vs Control, EA vs Model) in the estrogen signaling pathway and transcriptional misregulation in cancer signaling pathway.

**Table 1:** Expressions of reverted differentially expressed genes (DEGs) in pathways.

Estrogen signaling pathway				Transcriptional misregulation in cancer			
Gene	EA vs model regulate	Gene	Model vs control regulate	Gene	EA vs model regulate	Gene	Model vs control regulate
Shc3	up	Shc3	down	Aff1	down	Aff1	up
Pik3r3	up	Pik3r3	down	Myc	down	Myc	up
Ncoa3	down	Ncoa3	up	Ddit3	down	Ddit3	up
Jun	down	Jun	up	Cebpe	up	Cebpe	down
Hspa8	down	Hspa8	up	Kmt2a	down	Kmt2a	up
Hsp90ab1	down	Hsp90ab1	up	Plat	down	Plat	up
Hsp90aa1	down	Hsp90aa1	up	Fus	up	Fus	down
Gabbr2	up	Gabbr2	down	Nfkbiz	down	Nfkbiz	up
Fkbp5	down	Fkbp5	up	Cebpb	down	Cebpb	up
Fkbp4	down	Fkbp4	up	Polk	down	Polk	up

#### 3.2.4 Target analysis of pathway-associated reverted genes

To identify candidate pathway-associated genes regulated by EA, reverted DEGs from estrogen signaling pathway and transcriptional misregulation in cancer pathway were analyzed in Cytoscape by using CytoHubba with Maximal Clique Centrality (MCC) algorithm to score hub genes (**Figure 3F**, **Appendix Table 2**).

### 3.3 DIA-based proteomic analysis

#### 3.3.1 Proteomic profiling revealed differential proteins expression patterns

DIA-based proteomic analysis identified 69700 peptides and 6477 proteins, and the quantitative analysis results of DEPs between groups are shown in **Appendix Table 3**. Volcano plots (Appendix F1-A) and hierarchical clustering heatmaps (Appendix F1-B) demonstrated significant differences in protein expression profiles among three groups. The details of the proteome annotation database were shown in the **Supplementary Table 3**.

#### 3.3.2 GO enrichment and KEGG pathway analysis

Go functional enrichment analysis of DEPs was performed as shown in Appendix F1-C. In the Model vs. Control (MvsC) comparison, 398 GO terms were significantly enriched, including 286 biological processes (BPs). The EA vs. Model (EAvsM) comparison revealed 473 enriched GO terms, with 333 BPs. These DEPs were primarily associated with xenobiotic catabolic process, lipid metabolism process, hormone metabolic process, and cytoskeleton remodeling.

KEGG pathway analysis demonstrated that EA treatment significantly modulated 18 signaling pathways compared to the model group. These pathways were closely related to organic acid metabolism, immune regulation, steroid and cholesterol metabolism, and the cytochrome P450 enzyme system. Based on the KEGG enrichment results (*P* < 0.05), all significantly enriched pathways were visualized using bubble plots to illustrate their statistical and biological relevance (**Figure 4A**).

**Figure 4:**
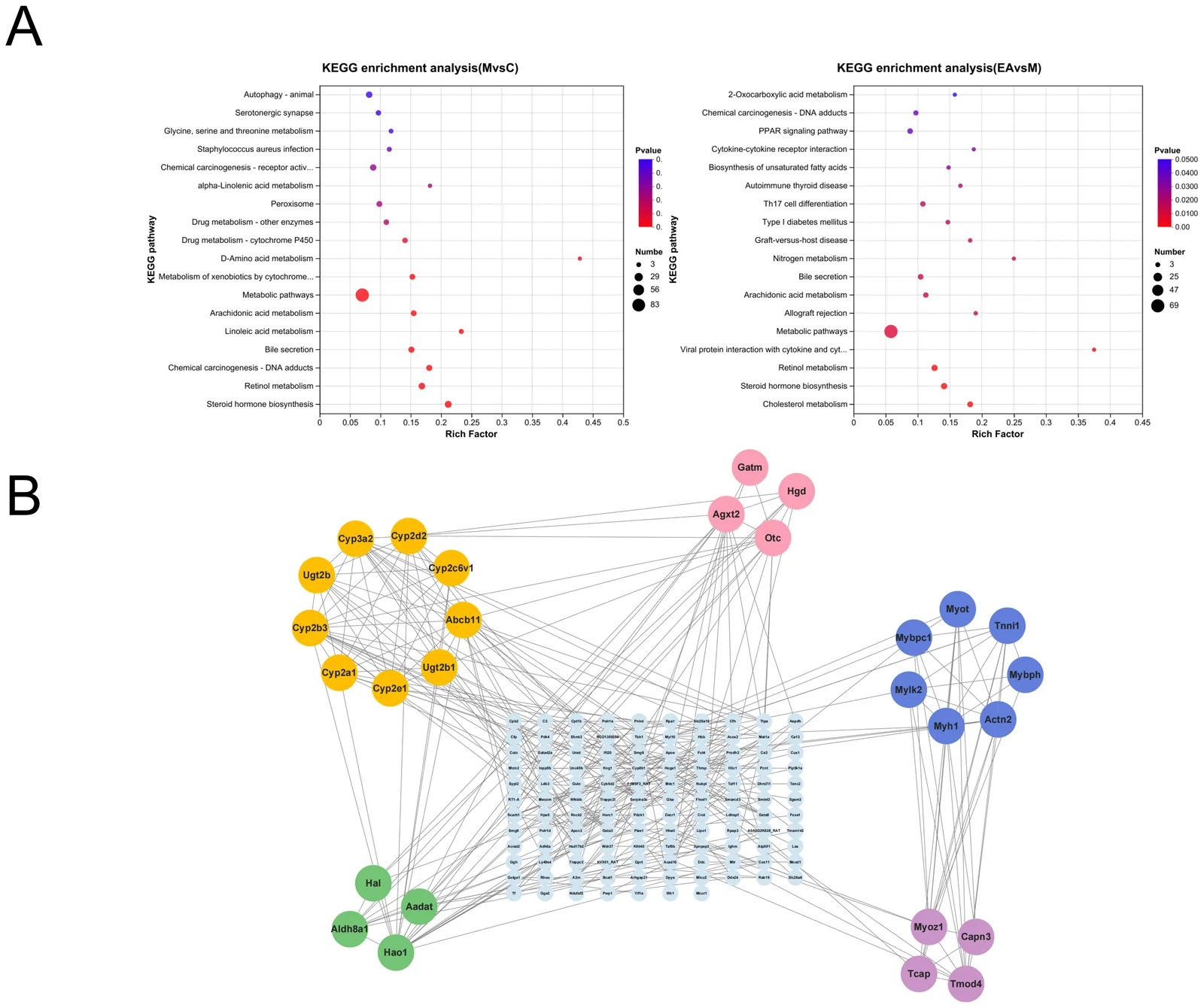
**(A)** KEGG enrichment analysis of differentially expressed proteins (DEPs) **(B)** MCODE Analysis of differentially expressed proteins (DEPs). (Cluster selection criteria: Degree≥2, remove single-connected nodes (Haircut), node score cutoff=0.2, K-Core=2, Max depth=100).

#### 3.3.3 MCODE analysis of differentially expressed proteins

Analysis of DEPs between EA and model groups using the MCODE plugin (Molecular Complex Detection) in Cytoscape identified five highly interconnected functional modules (node count>4), including drug metabolism (e.g., CYP2E1), amino acid metabolism (e.g., HAO1), muscle contraction (e.g., MYLK2), muscle regulation (e.g., TMOD4), and mitochondrial metabolism (e.g., GATM) as shown in **Figure 4B**.

### 3.4 Integrated transcriptomics and proteomics analysis

#### 3.4.1 Integrated transcriptome-proteome profiling identifies EA-responsive targets

A total of 6477 proteins were mapped to their encoding genes, and 429 molecules with concordant expression trends across the transcriptomic and proteomic datasets were identified by systematically comparing transcriptomic and DIA proteomic profiles in rat gastric tissues (**Figure 5A**).

**Figure 5:**
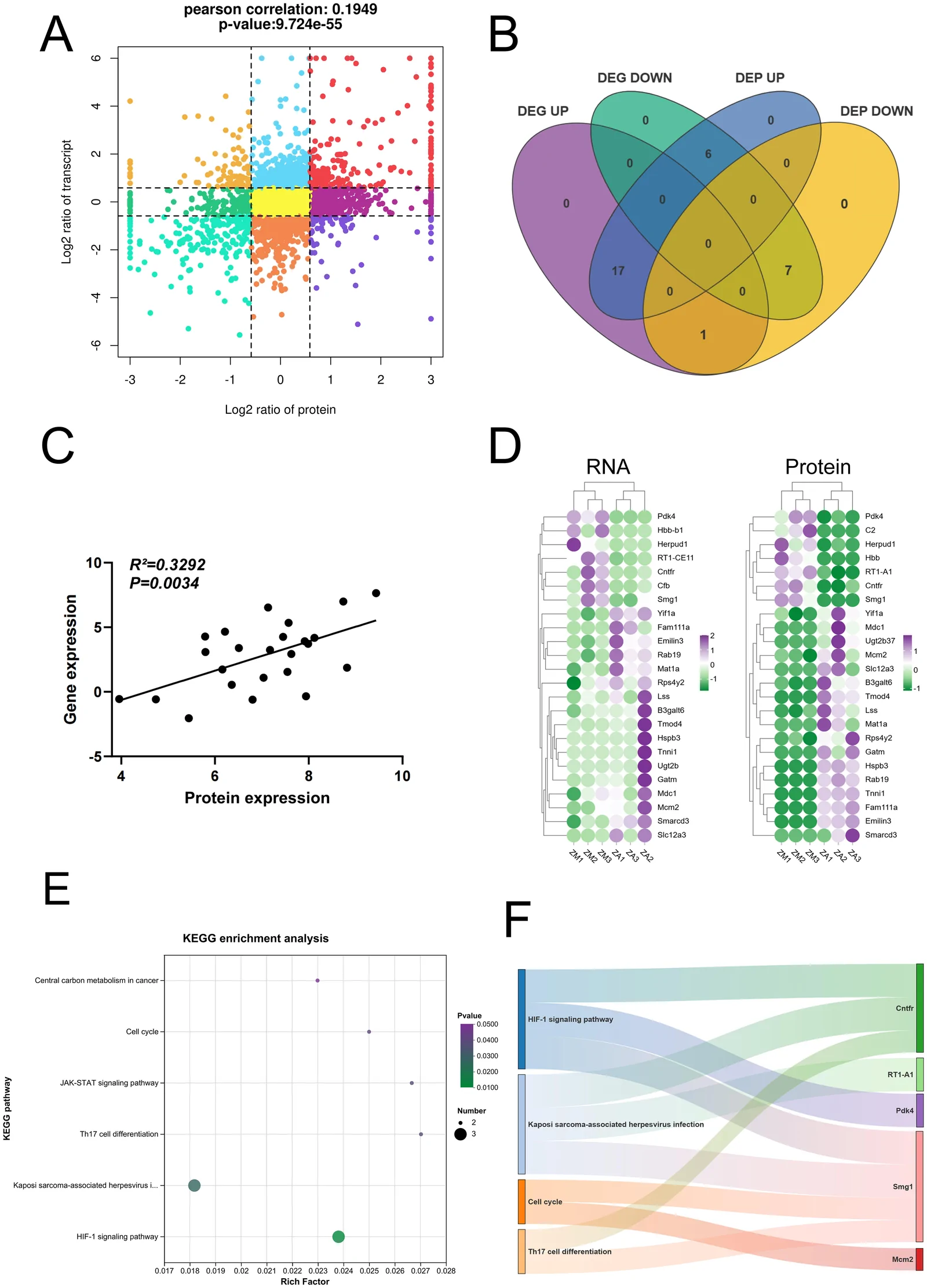
Integrated Transcriptomics and Proteomics Analysis. **(A, B)** Correlation analysis of differentially expressed genes/proteins in the comparison of EA group and Model group. [**(A)** Quadrant plots of differentially expressed genes/proteins; **(B)** Venn diagrams of differentially expressed genes/proteins. FC = 1.5, *P* < 0.05]. **(C, D)** Correlation Analysis and Heatmaps of consistent DEGs and DEPs [**(C)** Linear regression analysis of the 24 consistent DEGs and DEPs, **(D)** transcriptomic and proteomic clustering heatmaps of consistent genes/proteins] **(E)** KEGG analysis of consistent DEGs **(F)** Morula plot of enriched pathways and associated differentially expressed genes (DEGs).

Further analysis revealed 24 DEGs and DEPs with consistent changes in the comparison of EA and model group (**Figure 5B**), comprising 17 upregulated (B3GALT6, MCM2, EMILIN3, FAM111A, GATM, HSPB3, LSS, MAT1A, MDC1, RAB19, SLC12A3, SMARCD3, TMOD4, TNNI1, UGT2B, YIF1A, RPS4Y2) and 7 downregulated (CNTFR, PDK4, RT1-CE11, SMG1, CFB, HBB-B1, HERPUD1). These consistent DEGs and DEPs showed significant correlation between transcriptomics and proteomics (r^2^ = 0.329, *P* = 0.0034). The hierarchical clustering heatmaps showed that the expression patterns of consistent DEGs and DEPs in both transcriptomics and proteomics had significant differences between EA group and model group, as shown in **Figures 5C, D** and **Table 2**.

**Table 2:** Differential expression patterns of the 24 consistent DEPs in transcriptomic and proteomic profiles.

Gene id	Symbol	Proteomics		Transcriptomics	
		Log2FC	*P* value	Log2FC	*P* value
ENSRNOG00000019979	B3GALT6	3.14	0.02617	0.70	0.042858077
ENSRNOG00000047307	CNTFR	-1.99	0.02139	-3.16	0.004580469
ENSRNOG00000018796	HERPUD1	-1.57	0.0183	-2.00	0.0148138660264
ENSRNOG00000054549	LSS	0.82	0.0343	1.19	0.003323329
ENSRNOG00000016316	MCM2	0.94	0.04757	0.91	0.004638725
ENSRNOG00000009565	PDK4	-1.84	0.008099	-5.29	1.59E-19
ENSRNOG00000031041	RPS4Y2	1.15	0.021	0.98	0.002479292
ENSRNOG00000038999	RT1-CE11	-1.27	0.003734	-1.49	5.14E-09
ENSRNOG00000020201	YIF1A	0.78	0.02708	1.05	0.033196468
ENSRNOG00000051235	CFB	-16.61	0.003813	-2.22	5.80E-05
ENSRNOG00000016734	EMILIN3	7.28	0.0001816	1.85	0.0044726
ENSRNOG00000012067	FAM111A	7.28	0.0001834	4.74	9.68E-07
ENSRNOG00000000168	GATM	7.28	0.007531	1.54	0.004210293
ENSRNOG00000047098	HBB	-16.61	0.0008955	-3.75	0.00031271956773
ENSRNOG00000010992	HSPB3	7.28	7.846E-5	5.77	0.006351577
ENSRNOG00000011351	MAT1A	7.28	0.04032	2.00	0.000293146
ENSRNOG00000032813	MDC1	7.28	0.004206	0.94	0.004880652
ENSRNOG00000009030	RAB19	7.28	0.000217	2.05	0.008464622
ENSRNOG00000057072	SLC12A3	7.28	0.037	2.08	0.002133105
ENSRNOG00000010077	SMARCD3	7.28	0.04885	0.89	0.025612135
ENSRNOG00000047386	SMG1	-16.61	0.001694	-0.76	0.002735122
ENSRNOG00000021088	TMOD4	7.28	0.0003632	2.42	0.00444457
ENSRNOG00000009073	TNNI1	7.28	2.488E-5	5.62	2.74E-06
ENSRNOG00000046540	UGT2B37	7.28	0.01112	7.54	0.00063138

#### 3.4.2 KEGG enrichment analysis

KEGG enrichment analysis of consistent DEGs (screening criteria: *P* < 0.05, ≥2 proteins per pathway) revealed six significantly enriched pathways in the EA vs. model comparison **Figure 5D**): HIF-1 signaling pathway, Kaposi sarcoma-associated herpesvirus infection, central carbon metabolism in cancer, cell cycle, JAK-STAT signaling pathway, and Th17 cell differentiation pathway.

Among them, the pathways consistent with the enrichment of proteomics were Th17 cell differentiation (rno04659), and the pathways consistent with the enrichment of transcriptomics were HIF-1 signaling pathway (rno04066), Kaposi sarcoma-associated herpesvirus infection (rno05167), and cell cycle (rno04110). The enrichment pathways and the DEGs in the pathways are shown in **Figure 5F**.

### 3.5 RT-qPCR-based transcript-level assessment of PDK4 and SMG1

The PDK4 and SMG1, which showed a high expression consistency in protein and gene expressions (*P* < 0.01) and were related to CAG demonstrated in literatures, were selected for RT-qPCR-based transcript-level assessment based on their concordant expression trends and potential relevance to CAG. The RT-qPCR primer sequences of PDK4 and SMG1 were shown in the **Supplementary Table 4**. RT-qPCR analysis showed that the expression level of SMG1 in the model group was slightly increased compared to the control group, while the EA group exhibited a decreasing trend, with no statistically significant difference observed (*P >*0.05). PDK4 was significantly upregulated in model group compared with the control group (*P* < 0.01), and EA treatment markedly downregulated its expression (*P* < 0.01). This was consistent with the results of RNA-seq profiles (**Appendix Table 4**; **Figure 6**).

**Figure 6:**
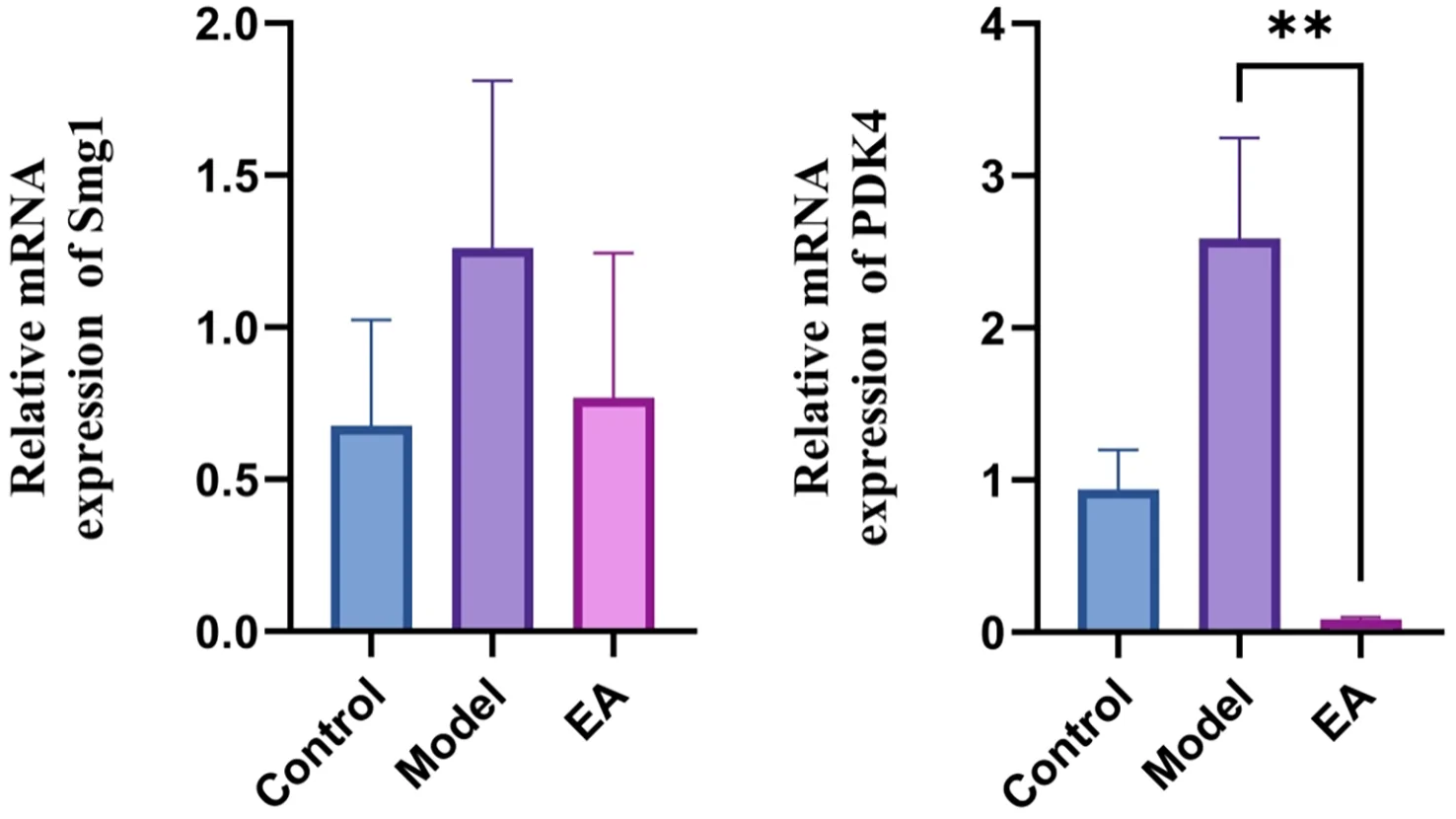
Relative expressions of SMG1 mRNA and PDK4 mRNA (**P < 0.01: versus the model group).

## 4 Discussion

Single-omics enrichment analysis (e.g., KEGG) presents inherent limitations: individual KEGG pathways may encompass multiple distinct biological processes wherein dysregulation of any constituent process could yield pathway enrichment or abundance elevation. When multiple genes/proteins within a pathway exhibit expression alterations due to abnormalities across different biological processes, even if such abnormalities do not directly drive a specific phenotype, the pathway may still show statistical significance in enrichment analyses (20). Furthermore, pathways frequently display mixed regulatory patterns with concurrent up- and down-regulation of components, rendering enrichment results alone insufficient for inferring overall biological relevance. Therefore, to mitigate overinterpretation of single-omics findings and enhance mechanistic understanding of EA in CAG treatment, we integrated transcriptomics and proteomics to identify consistently regulated genes/proteins in this study.

In the transcriptomic part, we employed MCODE analysis on the DEGs to identify and prioritize high-density subnetworks, which represent functionally closely-related gene modules. Subsequently, Cytohubba analysis was applied to DEGs within enriched pathways to pinpoint hub genes. By leveraging this strategy from network modules to hub genes, key regulatory genes can be identified with enhanced precision at the transcriptomic level. In the proteomics analysis, the MCODE algorithm was used to conduct modular analysis on the PPI network, the top five clustered protein modules were screened to decode the regulatory characteristics of EA at the functional module level. And then, integrated transcriptomic-proteomic analysis was conducted to elucidate precise candidate targets underpinning EA’s efficacy against CAG.

This study identified 506 significantly reversed DEGs following EA intervention. Core regulatory genes (Hspa8, Jun, Fkbp4, Fkbp5, and Hsp90aa1) formed a highly interactive network. Notably, these core genes exhibited significant enrichment in the estrogen signaling pathway (21). The loss of the Hsp90aa1 is associated with favorable outcomes after gastric cancer surgery (22), and Hsp90 inhibitors have been widely applied in the clinical treatment of various cancers. Fkbp4 and Fkbp5 serve as Hsp90-associated co-chaperones that can act as tumor-specific antigens to trigger immune responses (23). Mechanistically, c-Jun (a key AP-1 component regulating proliferation and stress response) is activated through Hsp90-mediated p38 phosphorylation (24), which concurrently upregulates Hsp90aa1 expression (25). On the other hand, c-Myc overexpression during CAG progression associates with inflammation, metaplasia and dysplasia (26) and its direct suppression induces tumor regression (27) Our data revealed that EA can significantly downregulates c-Myc expression, and this regulatory mode is consistent with the down-regulation trend of CDK4 and CDK6 after EA intervention. These findings suggest that downregulation of HSP90-related chaperones, together with altered expression of Myc and Jun, may be involved in EA-associated modulation of cellular stress responses and epithelial homeostasis in CAG.

Compared with transcriptomic analysis, proteomic analysis reflects dynamic functional protein alterations more directly. Unlike the transcriptomic patterns, PPI networks of reversed DEPs lacked significant clustering. To elucidate EA’s proteome-level regulatory mechanisms in CAG, we performed MCODE module mining on DEPs networks (28) Notably, EA significantly upregulated CYP2E1 protein expression in proteomic analysis. Previous studies have reported an association between CYP2E1 and advanced precancerous gastric lesions (29). Given that CYP2E1 is involved in oxidative metabolism and reactive oxygen species (ROS) generation, and may influence endoplasmic reticulum (ER) stress-related processes, its altered expression may reflect a complex adaptive response under chronic injury conditions rather than a unidirectional protective effect (30) Therefore, the present data are insufficient to support a specific mechanistic interpretation based on CYP2E1 alone. In addition, CYP2E1 was identified from proteomic module analysis rather than from the concordant multi-omics candidates selected for targeted validation, and its biological significance may vary across disease models and therapeutic contexts. Similarly, other proteins identified in the proteomic modules, including the muscle-related proteins MYLK2, MYBPC1, TNNI1, and MYOT, as well as the inflammation-associated protein HAO1, may be related to muscle-associated or inflammatory responses in gastric tissue. However, their precise roles in EA-associated molecular responses in CAG remain to be clarified. Collectively, these proteins should be interpreted as exploratory proteomic signals associated with EA intervention rather than validated mechanistic mediators.

Through integrated analysis of RNA-seq and DIA proteomics, we identified 24 genes/proteins with consistent expression trends. These consistent genes/proteins may represent candidate molecular targets associated with EA intervention in CAG. Notably, PDK4 displayed significant downregulation at both transcriptomic and proteomic levels after EA intervention, with expression reverting toward normal control levels. This reversal was also supported by qPCR analysis, further supporting PDK4 as a candidate molecule associated with the EA response.

PDK4 (pyruvate dehydrogenase kinase 4) is a key enzyme that regulates glucose metabolism and mitochondrial function. By inhibiting the expression of PDK4, the phenotype can be promoted to shift towards a pre-glycolytic state (31), and the architecture and function of mitochondria can be restored (32). Unlike other PDK subtypes, PDK4 exhibits dual functions in tumors: it not only has oncogenic functions but also tumor suppressor functions (33). It has been reported that targeting PDK4 can block glycolysis, inhibit and delay the occurrence of liver cancer (34). PDK4 is an important regulator of cellular energy metabolism. In the present study, EA intervention was associated with reduced PDK4 expression, suggesting that PDK4 may be involved in the response to EA in CAG. Based on previous studies, changes in PDK4 may be related to alterations in the metabolic state of gastric mucosal cells. In this study, we also measured CDK4 and CDK6 expressions, given their important role in cell cycle regulation and proliferation (35, 36). CDK4 and CDK6 are key regulators of G1/S cell-cycle progression, and their aberrant activation is closely associated with dysregulated cell proliferation. Enhanced epithelial proliferation in CAG is implicated in premalignant progression and mucosal damage. Aberrant activation of the CyclinD-CDK4/6-Rb axis drives malignant proliferation (37, 38). It has been confirmed that cyclin-dependent kinase (CDK)4 and CDK6 are frequently overexpressed or hyperactivated in human cancers (39). Small-molecule CDK4/6 inhibitors effectively enhanced focal adhesion kinase (FAK) inhibitor efficacy for gastric cancer *in vitro* and *in vivo (*40). In this study, we found that compared with the control group, both CDK4 and CDK6 mRNA levels were significantly upregulated in the model group, and EA intervention could downregulate CDK4 and CDK6 expressions significantly. These findings suggest that EA may be associated with the modulation of cell-cycle-related molecular changes. Together with previous reports, these findings suggest that alterations in CDK4/CDK6 may be involved in the regulation of abnormal proliferative activity in CAG gastric mucosa. The findings of this study should be interpreted in the context of several considerations. First, although *Helicobacter pylori* is a major etiological factor in chronic atrophic gastritis, the present study employed a non-*H. pylori*, multifactorial injury model; therefore, our results mainly reflect EA-associated changes in this specific experimental context and should be extrapolated to *H. pylori*-related CAG with caution. Second, the absence of a sham-acupuncture or non-acupoint stimulation group made it difficult to distinguish acupoint-specific effects from non-specific somatosensory stimulation. Third, CDK4/6 and selected omics-derived candidates were assessed mainly at the transcript level, without additional immunohistochemical, protein-level or gain-/loss-of-function validation. Accordingly, PDK4, CDK4, and CDK6 should be regarded as candidate molecules associated with the EA response rather than definitive causal mediators. In addition, several proteins identified from proteomic module analysis, including CYP2E1 and other module-associated molecules, were derived from exploratory network-based screening rather than from the concordant multi-omics candidates selected for targeted validation. Their biological significance may vary across disease models and therapeutic contexts; therefore, the altered expression of these proteins should be interpreted cautiously and considered candidate molecular associations that warrant further validation. Finally, this study assessed gastric tissue changes immediately after a 2-week EA intervention, and the persistence of these effects after treatment cessation remains unclear. Despite these limitations, the present study provides an exploratory multi-omics framework for understanding EA-associated molecular changes in CAG. Future studies incorporating H. pylori-related models, sham controls, longer follow-up time points, and protein- and function-level validation will help further clarify the specificity and mechanistic relevance of these findings.

## 5 Limitations

The present study was designed to investigate the therapeutic response and associated molecular alterations induced by a predefined EA intervention, rather than to dissect the acupoint-specific effects or the independent contributions of needling and electrical stimulation. Therefore, the CAG model group served as the primary disease control, and additional sham-EA or non-acupoint EA groups were not included. Nevertheless, the absence of these control groups prevents us from distinguishing acupoint-specific effects from those attributable to needling, electrical stimulation, restraint, or other nonspecific components of the intervention. Future studies incorporating appropriately designed sham-EA and non-acupoint controls are therefore warranted.

Histopathological evaluation in the present study was primarily qualitative and did not include quantitative or semi-quantitative scoring. Given the potential regional heterogeneity of gastric mucosal lesions, future studies should incorporate standardized tissue sampling together with blinded histopathological scoring to improve the objectivity and reproducibility of pathological assessment.

## Data Availability

The mass spectrometry proteomics raw data have been deposited to the ProteomeXchange Consortium via the PRIDE repository with the dataset identifier PXD084030. The RNA-seq raw sequencing data have been deposited in the NCBI Sequence Read Archive (SRA) under BioProject accession PRJNA1452024.
