# Supplementary material for: Integrated transcriptomic and proteomic profiling suggests PDK4 and cell cycle-related responses to electroacupuncture in a rat model of chronic atrophic gastritis
Source: Front Gastroenterol (Lausanne). 2026 Oct 30;5:1894243. doi: 10.3389/fgstr.2026.1894243 (PMC13759280; doi:10.3389/fgstr.2026.1894243)
Supplement: Supplementary file 1 [file Table1.docx]

**Supplementary Tables**

**Supplementary Table 1. Weight changes of rats in each group (**$\bar{\boldsymbol{x}}\boldsymbol{\pm}\boldsymbol{s}$**, g)**

| Time | Control group | Model group | EA group |
| --- | --- | --- | --- |
| 1 | 267.06±14.24 | 247.36±12.06^*^ | 244.43±14.97^#^ |
| 2 | 319.54±10.57 | 276.41±9.72^**^ | 292.33±21.50^##^ |
| 3 | 368.33±16.97 | 307.91±9.14^**^ | 308.17±19.06^##^ |
| 4 | 410.79±21.51 | 316.99±13.17^**^ | 321.09±17.54^##^ |
| 5 | 423.53±23.91 | 346.62±18.84^**^ | 355.79±21.36^##^ |
| 6 | 438.21±27.48 | 381.38±18.28^**^ | 381.90±25.67^##^ |
| 7 | 464.39±27.50 | 388.41±20.12^**^ | 378.071±26.170^##^ |
| 8 | 485.56±29.64 | 425.39±22.85^**^ | 404.343±29.16^##^ |
| 9 | 507.44±34.98 | 450.16±23.75^*^ | 433.514±39.99^##^ |
| 10 | 502.09±16.60 | 426.13±23.69^**^ | 412.386±36.90^##^ |
| 11 | 536.60±38.08 | 465.34±27.314^**^ | 443.000±43.80^##^ |
| 12 | 531.66±38.24 | 467.14±31.08^**^ | 448.143±43.50^##^ |

Note: * P<0.05，** P<0.01: versus the control group； #P<0.05，## P<0.01: versus the model group.

**Supplementary Table 2-1. Transcriptome sequencing data**

| **Sample** | **Raw reads** | **Raw bases** | **Clean reads** | **Clean bases** | **Error rate**  **(%)** | **Q30**  **(%)** | **GC content(%)** |
| --- | --- | --- | --- | --- | --- | --- | --- |
| ZC1 | 44426816 | 6708449216 | 43949520 | 6582682084 | 0.0256 | 94.91 | 51.01 |
| ZC2 | 43891940 | 6627682940 | 43413208 | 6506966991 | 0.0257 | 94.87 | 50.68 |
| ZC3 | 45145264 | 6816934864 | 44614156 | 6688240241 | 0.0257 | 94.83 | 50.33 |
| ZM1 | 45679812 | 6897651612 | 45162184 | 6772995377 | 0.0255 | 94.97 | 50.35 |
| ZM2 | 47871174 | 7228547274 | 47312454 | 7086051363 | 0.0257 | 94.82 | 49.26 |
| ZM2 | 43991820 | 6642764820 | 43504942 | 6524904470 | 0.0256 | 94.91 | 49.56 |
| ZA1 | 44847302 | 6771942602 | 44333862 | 6643616205 | 0.0258 | 94.8 | 50.83 |
| ZA2 | 45682048 | 6897989248 | 45203348 | 6779731905 | 0.0255 | 95.03 | 49.61 |
| ZA3 | 48734366 | 7358889266 | 48174744 | 7201883932 | 0.0254 | 95.04 | 51.98 |

**Supplementary Table 2-2. Comparison of transcriptome sequence**

| **Sample** | **Total reads** | **Total mapped** | **Multiple mapped** | **Unique mapped** |
| --- | --- | --- | --- | --- |
| ZC1 | 43949520 | 42877069(97.56%) | 3239105(7.37%) | 39637964(90.19%) |
| ZC2 | 43413208 | 42342071(97.53%) | 2990937(6.89%) | 39351134(90.64%) |
| ZC3 | 44614156 | 43443101(97.38%) | 2220121(4.98%) | 41222980(92.4%) |
| ZM1 | 45162184 | 44071838(97.59%) | 2676183(5.93%) | 41395655(91.66%) |
| ZM2 | 47312454 | 46079802(97.39%) | 1832001(3.87%) | 44247801(93.52%) |
| ZM3 | 43504942 | 42282088(97.19%) | 1581301(3.63%) | 40700787(93.55%) |
| ZA1 | 44333862 | 43224967(97.5%) | 2720137(6.14%) | 40504830(91.36%) |
| ZA2 | 45203348 | 44058203(97.47%) | 1522261(3.37%) | 42535942(94.1%) |
| ZA3 | 48174744 | 47040808(97.65%) | 3817706(7.92%) | 43223102(89.72%) |

**Supplementary Table 3. Proteome Annotation Database**

| **Annotation database** | **Number of Annotations** | **Total Protein Count** | **Annotation Ratio** | **e_value** |
| --- | --- | --- | --- | --- |
| **NR** | 6477 | 8218 | 100% | / |
| **SubCell-Location** | 6477 | 8218 | 100% | / |
| **Transcription-Factors** | 115 | 8218 | 1.78% | / |
| **EggNOG** | 6451 | 8218 | 99.60% | / |
| **GO** | 6234 | 8218 | 96.25% | 1.00E-05 |
| **KEGG** | 6469 | 8218 | 99.88% | 1.00E-05 |
| **Pfam** | 6293 | 8218 | 97.16% | 1.00E-05 |

**Supplementary Table 4.** **RT-qPCR primer sequences of PDK4 and SMG1**

| **Target Gene** | **Sequence (5'-3')** | **Length (bp)** |
| --- | --- | --- |
| **R-PDK4-F** | GACATCCTCCCTGAACGCTTAGT | 118 |
| **R-PDK4-R** | CTTTCTGGTCTTCTGGGCTCTTT |  |
| **R-SMG1-F** | ATGTCTGCCGTGTTCAGCTTGT | 179 |
| **R-SMG1-R** | AGGGTGGAACGTGTTACTTGGT |  |
| **R-GAPDH-F** | CTGGAGAAACCTGCCAAGTATG | 138 |
| **R-GAPDH-R** | GGTGGAAGAATGGGAGTTGCT |  |
